# Supplementary material for: Three clusters of content-audience associations in expression of racial prejudice while consuming online television news
Source: PLoS One. 2021 Jul 23;16(7):e0255101. doi: 10.1371/journal.pone.0255101 (PMC8301668; doi:10.1371/journal.pone.0255101)
Supplement: S1 Table — The total number of all comments mentioning Koreans was 81,114. (PDF) [file pone.0255101.s001.pdf]

S1 Table: Number of audiences/news grids/comments of each cluster of modern/old-fashioned racism. The total number of all comments mentioning Koreans was 81,114.

	Modern racism			Old-fashioned racism		
	Audience	News Grid	Comment	Audience	News Grid	Comment
Ambiguous	868 (25.5%)	1,214 (16.4%)	1,719 (16.9%)	2,230 (65.6%)	3,349 (45.2%)	4,860 (47.9%)
Evocative	484 (14.2%)	1,006 (13.6%)	1,120 (11.0%)	505 (14.9%)	889 (12.0%)	1,029 (10.1%)
Non-evocative	20 (0.589%)	628 (8.15%)	628 (5.19%)	346 (10.2%)	1022 (13.8%)	1,028 (10.2%)
Non-expression	2,185 (64.3%)	4,555 (61.5%)	6,684 (65.8%)	642 (18.9%)	2,143 (28.9%)	3,234 (31,9%)
Total	3,398 (100.0%)	7,403 (100.0%)	10,151 (100.0%)	3,398 (100.0%)	7,403 (100.0%)	10,151 (100.0%)
