# Supplementary material for: Three clusters of content-audience associations in expression of racial prejudice while consuming online television news
Source: PLoS One. 2021 Jul 23;16(7):e0255101. doi: 10.1371/journal.pone.0255101 (PMC8301668; doi:10.1371/journal.pone.0255101)
Supplement: S2 Table — (PDF) [file pone.0255101.s002.pdf]

S2 Table: Frequencies and ratios of audiences, grids, and comments associated to modern/old-fashioned racism

	Audiences	News Grid	Comments
Only Modern Racism	642 (18.9%)	2,143 (28.9%)	3,234 (31.9%)
Only Old-fashioned Racism	2,185 (64.3%)	4,555 (61.5%)	6,684 (65.9%)
Both	571 (16.8%)	705 (9.5%)	223 (2.2%)
Total	3,398 (100.0%)	7,403 (100.0%)	10,151 (100.0%)
