# Supplementary material for: Three clusters of content-audience associations in expression of racial prejudice while consuming online television news
Source: PLoS One. 2021 Jul 23;16(7):e0255101. doi: 10.1371/journal.pone.0255101 (PMC8301668; doi:10.1371/journal.pone.0255101)
Supplement: S3 Table — Columns and rows show modern/old-fashioned racism, respectively. The interclass correlation between both racism clusters was negative (Cohen’s κ = −0.332 where p-value was less than 10−16). Diagonal elements were also relatively small. The news grids where audiences did not express any racism were not included in the dataset of this study, i.e., the cluster size of non-expression of modern and old-fashioned racism was zero. (PDF) [file pone.0255101.s003.pdf]

S3 Table: Co-occurrences of news grid clusters between modern/old-fashioned racism. Columns and rows show modern/old-fashioned racism, respectively. The interclass correlation between both racism clusters was negative (Cohen's $\kappa = -0.332$ where p-value was less than 10^{-16}). Diagonal elements were also relatively small. The news grids where audiences did not express any racism were not included in the dataset of this study, i.e., the cluster size of non-expression of modern and old-fashioned racism was zero.

Old-fashioned / Modern	Ambiguous	Evocative	Non-evocative	Non-expression	Total
Ambiguous	248	153	70	2,878	3,349
Evocative	60	44	23	762	889
Non-evocative	52	40	15	915	1,022
Non-expression	854	769	520	0	2,143
Total	1,214	1,006	628	4,555	7,403
