# Supplementary material for: Three clusters of content-audience associations in expression of racial prejudice while consuming online television news
Source: PLoS One. 2021 Jul 23;16(7):e0255101. doi: 10.1371/journal.pone.0255101 (PMC8301668; doi:10.1371/journal.pone.0255101)
Supplement: S2 Dataset — (RTF) [file pone.0255101.s006.rtf]

# * is wild card.
�Ý“ú•Ä (Zainichi-Bei): Americans in Japan. Often refer to American residents in Japan or U.S. army stationed in Japan
�Ý“úƒAƒ�ƒŠƒJ (Zainichi-Amerika): Same as Zainichi-Bei.
�Ý“ú*‘åŽgŠÙ (Zainichi-Taishikan): embassy of * in Japan
�Ý“úƒvƒ‰ƒWƒ‹(Zainichi-Burajiru): Brazilian in Japan
�Ý“ú’†�‘ (Zainichi-Chugoku): Chinese in Japan
�Ý“úƒyƒ‹�[ (Zainichi-Peru): Peruvian in Japan
�Ý“úƒtƒ@ƒ“ƒN(Zainichi-Fanku): name of a funk band
’©‘N�lŽQ (chosen-ninjin): Asiatic ginseng
ƒ`ƒ‡ƒ“�Ø (chongiru): chop off
Žñƒ`ƒ‡ƒ“ (kubi-chon): cut somebody's head off 
ƒ`ƒ‡ƒ“ƒp (chonpa): an onomatopoeia
ƒNƒGƒXƒ`ƒ‡ƒ“ (kueschon): question
ƒ`ƒ‡ƒ“ƒK�[ (chonga): unmarried men
ƒ`ƒ‡ƒ“ƒ{ (chonbo): goof
ƒ`ƒ‡ƒ“ƒ}ƒQ (chonmage): topknot
ƒ`ƒ‡ƒ“*ƒEƒ\ƒ“ (Chon Uson): a person's name
ƒ`ƒ‡ƒ“*ƒ_ƒ€ƒhƒ“ (Chon Damudon): a person's name
ƒ`ƒ‡ƒ“*ƒTƒKƒ“ (Chon Sagan):  a person's name
ƒ`ƒ‡ƒ“*ƒ_ƒ“ƒr (Chon Danbi): a person's name
ƒ`ƒ‡ƒ“*ƒWƒqƒ‡ƒ“ (Chon Jihyon): a person's name
ƒ`ƒ‡ƒ“*ƒ†ƒ~ (Chon Yumi) : a person's name
ƒ`ƒ‡ƒ“*ƒwƒCƒ“ (Chon Hein): a person's name
ƒ`ƒ‡ƒ“*ƒ_ƒˆƒ“ (Chon Dayon): a person's name
ƒ`ƒ‡ƒ“*ƒxƒbƒLƒ‡ƒ“ (Chon Bekkyon): a person's name
ƒ`ƒ‡ƒ“*ƒqƒ‡ƒN (Chon Hyoku): a person's name
ƒ`ƒ‡ƒ“*ƒ_ƒrƒ“(Chon Dabin): a person's name
ƒ`ƒ‡ƒ“*ƒCƒ€ (Chon Imu): a person's name
ƒ`ƒ‡ƒ“*ƒE (Chon U): a person's name
ƒ`ƒ‡ƒ“*ƒEƒ“ƒCƒ“ (Chon Un'in): a person's name
ƒ`ƒ‡ƒ“*ƒ}ƒlƒˆ (Chon Maneyo): a person's name
ƒ`ƒ‡ƒ“*ƒ}ƒŠƒˆ (Chon Mariyo): a person's name
ƒ`ƒ‡ƒ“*ƒXƒ“ƒW (Chon Sunji): a person's name
ƒ`ƒ‡ƒ“*ƒWƒ…ƒm (Chon Jyuno): a person's name
ƒ`ƒ‡ƒ“*ƒCƒ‹ƒˆƒ“ (Chon Iruyon): a person's name
ƒ`ƒ‡ƒ“*ƒ}ƒ‹ (Chon Maru): a person's name
ƒ`ƒ‡ƒ“*ƒ„ƒMƒ‡ƒ“ (Chon Yagyon): a person's name
ƒ`ƒ‡ƒ“‚ÖƒCƒ“ (Chon Hein): a person's name
ƒ`ƒ‡ƒ“*ƒoƒL (Chon Baki): a person's name
ƒ`ƒ‡ƒ“*ƒ{ƒWƒ“ (Chon Bojin): a person's name
ƒ`ƒ‡ƒ“*ƒ\ (Chon So): a person's name
ƒ`ƒ‡ƒ“*ƒhƒˆƒ“ (Chon Doyon): a person's name
ƒ`ƒ‡ƒ“ƒX (Chonsu): a person's name
ƒ`ƒ‡ƒ“ƒjƒ‡ (Chonnyo): a person's name
ƒ`ƒ‡ƒ“ƒiƒ“ƒWƒ‡ƒ“ (Chon Nanjon): a person's name
ƒ`ƒ‡ƒ“ƒ} (Chonma): a person's name
ƒ`ƒ‡ƒ“ƒOƒ€ (Chongumu): a person's name
ƒ†ƒ`ƒ‡ƒ“(Yuchon): a person's name
ƒ„ƒ`ƒ‡ƒ“(Yachon): a person's name
ƒIƒNƒ`ƒ‡ƒ“ (Okuchon): a person's name�j
ƒ`ƒ‡ƒ“ƒz (Chonho): a name of a place in Korean
ƒCƒ`ƒ‡ƒ“ (Ichon): person's name
ƒ\ƒNƒ`ƒ‡ƒ“ (Sokuchon): a person's name
ƒzƒNƒ`ƒ‡ƒ“ (Hokuchon): a person's name
ƒRƒbƒ`ƒ‡ƒ“ (Kocchon): a person's name
ƒlƒ`ƒ‡ƒ“ (Nechon): a person's name
ƒXƒNƒ`ƒ‡ƒ“ (Sukuchon): a person's name
ƒO*ƒ`ƒ‡ƒ“ (Guchon): a person's name
ƒR*ƒ`ƒ‡ƒ“ƒ} (Ko Chonma): a person's name
ƒ`ƒ…ƒ“*ƒ`ƒ‡ƒ“ (Chun Chon): a person's name
ƒTƒ€*ƒ`ƒ‡ƒ“ƒ| (Samu Chonpo): a person's name
ƒ`ƒ‡ƒ“ƒ_ƒ€ƒAƒŠƒX (Chondamu Arisu): a name of Korean drama
