# Supplementary material for: Three clusters of content-audience associations in expression of racial prejudice while consuming online television news
Source: PLoS One. 2021 Jul 23;16(7):e0255101. doi: 10.1371/journal.pone.0255101 (PMC8301668; doi:10.1371/journal.pone.0255101)
Supplement: Attachment — Submitted filename: plosone_response.pdf [file pone.0255101.s013.pdf]

June 29th, 2021

Dear Editor,

I would like to thank the editors and the reviewers for carefully reading our manuscript and providing constructive and insightful comments. In response to the reviewers' comments, we have revised the manuscript "Three clusters of content-audience associations in expression of racial prejudice while consuming online television news " (PONE-D-20-34442R1). We look forward to the publication of our manuscript in PLOS ONE. We have improved the paper by responding to all the reviewers' comments. In the revised manuscript, the changes appear in red text for easy identification. The corresponding reviewers' comments of the suggested changes are shown in the right margins of the manuscript, e.g., "R1-2" means Reviewer 1's comment 2.

Sincerely,

**Masanori Takano, Ph.D. CyberAgent, Inc.**
**Akihabara Daibiru 13F, 1-18-13 Sotokanda**
**Chiyoda-ku, Tokyo 101-0021, Japan**
**Tel: +81-50-5578-0441**
**E-Mail: takano_masanori@cyberagent.co.jp**

# Response for the First Reviewer

## Comment 1

I believe the authors did an excellent job of revising this paper. The concepts are defined better (such as racism and hate speech) and there is more context for Japan for international readers (more could be done here). The added table is very good, summarising the findings. The part where the authors define racism towards the end of the 15th and the top of the 16th pages is good, but it concentrates a bit too much on McConahay's take on racism. I still think TV audiences should be conceptualised in the paper using existing audience research literature. Also, the paper would still benefit from a good read of its English: for example, imposing a time-pressures -15th page/Not romas but roma – 16th page. Also, I think this sentence should be removed: "However, we do not have intention to exclude conceptualizations other than McConahay's". Finally, on the 18th page, right before the title "results", there are some fragmented very short paragraphs – could be good if the authors turn them into better a better flowing longer paragraph. Looking forward to reading the article's published version.

### Response 1

Thank you for your positive evaluation of our revisions.

We have revised our manuscript based on your suggestion:

- We have described the relevance of this study to audience researches in the Introduction (p.2, L.42) and the Discussion (p.11, L.375) sections.
- We have carefully reviewed the manuscript and revised some expressions, including "a time-pressures" and "romas."
- According to your comment, we have removed the sentence "However, we do not have intention to exclude conceptualizations other than McConahay's."
- We have also changed the fragmented paragraphs right before the Results section in the following: 1) We have removed the sentence "Previous studies have shown that the expression of both modern and old-fashioned racism is frequent in Japanese social media, as cited above [7,32]" because there was a similar sentence (the 12th paragraph) in the Introduction (L.103) section; 2) we have moved the sentence "The antipathy toward Koreans by the Japanese can be partly attributed to extreme 177 tension between the two Korean states and Japan over historical and territorial 178 issues [36,58,61,62]" to the last part of the 21th paragraph (L.170) in the Introduction section.

# Response for the Second Reviewer

## Comment 1

I want to thank the editor and the authors for having given me the opportunity to review this manuscript a second time. I really believe that the authors tried to address all my previous comments. Generally, I do believe that the manuscript is now ready to publish. However, I would like them to clarify a little better what they did in the validation of the dictionary first. Particularly part 2 of their validation seems a bit ominous: "2) two trained coders independently evaluated each comment whether they match the definition of relevant form of racism". When validating the dictionaries, did you test whether it the respective racism dictionary discriminates against non-racist comments or against comments including the second type of racism? Judging from the high validity measures, I fear that they did the former. So, we don't really know how well it discriminates between the two types of racism. If it actually is the latter, I am a bit skeptical that the authors may have over-fitted the dictionary to their specific data. Otherwise all concerns have been addressed either by clarification or addressing the limitation in the text.

### Response 1

Thank you for your comments about the concerns over the validation process and dictionary over-fitting.

In the validation process, we classified the comments in view of one specific form of racism at once. In other words, the comments that did not refer to target racism but did to the other form of racism belonged to the contrast group among those did not refer to either form of racism. In addition, the dictionaries used in the present study were fixed before the data collection by minor revisioning of a previous study, and no retrospective adjustments to fit our specific dataset were conducted. Thus, the high accuracy measures is not a result of inadequate comprarisons or over-fitted dictionaries. To make this point clear, we have added the above description to the Dataset in the Materials and Methods (L.575 and L.582) section.
